# Psychosocial School Conditions and Mental Wellbeing Among Mid-adolescents: Findings From the 2017/18 Swedish HBSC Study

**DOI:** 10.3389/ijph.2022.1605167

**Published:** 2023-01-05

**Authors:** Sara Brolin Låftman, Bitte Modin, Maria Granvik Saminathen, Viveca Östberg, Petra Löfstedt, Kristiina Rajaleid

**Affiliations:** ^1^ Department of Public Health Sciences, Faculty of Social Sciences, Stockholm University, Stockholm, Sweden; ^2^ Department of Public Health and Community Medicine, Institute of Medicine, Sahlgrenska Academy, University of Gothenburg, Gothenburg, Sweden; ^3^ Stress Research Institute, Department of Psychology, Faculty of Social Sciences, Stockholm University, Stockholm, Sweden

**Keywords:** school demands, teacher support, classmate support, wellbeing, school

## Abstract

**Objectives:** To investigate mid-adolescent boys’ and girls’ experiences of school demands, teacher support, and classmate support, and explore the associations of these factors with mental wellbeing.

**Methods:** Data were derived from the Swedish Health Behaviour in School-aged Children (HBSC) study of 2017/18, with information collected among 1,418 students in grade 9 (∼15–16 years). School demands, teacher support, and classmate support were measured by indices based on three items each. Mental wellbeing was measured by the Short Warwick-Edinburgh Mental Wellbeing Scale (SWEMWBS). Linear regression analyses were performed.

**Results:** Higher demands were associated with lower mental wellbeing. Conversely, mental wellbeing increased with greater teacher support and classmate support. Interactions between demands and the support variables showed that at the lowest levels of teacher and of classmate support, mental wellbeing was low and not associated with school demands. With increasing levels of teacher and classmate support, the overall level of mental wellbeing increased and revealed an inverse association between school demands and mental wellbeing.

**Conclusion:** The study contributes with knowledge about how psychosocial conditions in school may hinder or enhance wellbeing among students.

## Introduction

School is an important context for children and young people where they spend a significant amount of time during their formative years. The school environment contains both stressors and resources that may affect children’s mental wellbeing. According to the Swedish Education Act [1], schools in Sweden are obliged to engage in both health prevention and promotion. While preventative efforts aim to fend off and protect against health adversities, health-promoting work revolves around strengthening the prerequisites for good health.

Although psychosocial school conditions may be linked to both adverse and positive health outcomes, previous research has largely focused on aspects of the former, such as depressive symptoms [2] or multiple health complaints, i.e., psychological complaints such as feeling sad or down, or feeling nervous, and somatic complaints such as headache and stomach-ache [3–12]. A more limited number of studies have assessed the links between psychosocial school conditions and aspects of students’ positive mental health. Yet, identifying school-related determinants of students’ mental wellbeing has the potential to support schools’ health-promoting work.

The concept of mental wellbeing can be understood in light of WHO’s definition of mental health as “a state of wellbeing in which an individual realises his or her own abilities, can cope with the normal stresses of life, can work productively and is able to make a contribution to his or her community.” [13] More specifically, mental wellbeing covers both a hedonic component (feelings of happiness and pleasure), and a eudaimonic one (feelings of meaning and self-realisation, and positive functioning) [14–17].

### Previous Research on Psychosocial School Conditions and Health Outcomes

Much prior research on psychosocial school conditions has applied a stress-theoretical perspective and studied aspects of the school environment in relation to various health problems. One commonly examined outcome is adolescents’ subjective health complaints, which can be seen as a marker for stress [18]. Studies have shown that students who experience high school demands tend to report higher levels of perceived stress [19], subjective health complaints [3–12, 19, 20], and conduct problems [21]. By contrast, supportive relations at school are inversely linked with health problems among students. Perceived teacher support has been shown to be associated with fewer depressive symptoms [2] and fewer health complaints [10, 12, 21–23]. Similarly, higher levels of classmate support have been shown to be linked with fewer health complaints [10, 21, 22, 24], whereas students with problematic classmate relations report more health complaints [3]. In line with the demand-control-support model, it has also been suggested that high control as well as high levels of social support may buffer against the association between school demands and health complaints [5, 10].

While a relatively large number of studies have addressed psychosocial school conditions in relation to various health problems, the links with salutogenic health outcomes are somewhat less explored. However, research has shown associations between different aspects of positive mental health and school pressure and demands [11, 20, 25] as well as classmate support [3, 11, 25, 26, 27], and teacher support [25–28]. To the best of our knowledge, however, there is a shortage of studies that simultaneously address students’ perceptions of school demands, teacher support, and classmate support in relation to measures of positive mental health (but see [25] as well as [29, 30] which were based on the same data as the current study). There is also a lack of studies examining if teacher and classmate support can moderate the association between school demands and mental wellbeing.

With regards to mental wellbeing, higher levels tend to be reported for boys than for girls [26, 31, 32]. Furthermore, girls tend to report higher school demands than boys [19, 21, 23, 28, 33]. While previous findings regarding gender differences in perceived teacher support are mixed [21, 23, 27], boys have been shown to report higher levels of peer support than girls [21, 27]. Studies have also indicated that the association between school demands and health outcomes is stronger among girls than among boys [2, 3, 19, 23, 34]. Concerning gender differences in the associations between social support and adolescent mental health outcomes, the evidence is however more mixed [21]. Taken together, it seems clear that gender needs to be taken into consideration when examining psychosocial school conditions and student mental wellbeing.

Socioeconomic status in terms of family affluence has been shown to be positively related with students’ mental wellbeing [14]. However, the international Health Behaviour in School-aged Children (HBSC) 2013/14 report concluded that there was no clear pattern by family affluence for school pressure or classmate support [35]. Nonetheless, other studies have shown that adolescents in families with economic hardship have fewer friends in the class [36, 37] and are more often socially isolated [37]. Students from economically vulnerable households have also reported less teacher support [38]. Hence, in an analysis of psychosocial school conditions and mental wellbeing, adjusting for family affluence is relevant.

### Aim of the Study

The aim of the present study was to investigate mid-adolescent boys’ and girls’ experiences of school demands, teacher social support, and classmate social support and the associations of these factors with mental wellbeing as captured through the Short Warwick-Edinburgh Mental Wellbeing Scale (SWEMWBS). A second aim was to examine if teacher social support and classmate social support modified the association between school demands and mental wellbeing.

## Methods

### Data Material

The data were derived from the Swedish HBSC study conducted among students in grades 5, 7, and 9 in the school year 2017/18. The HBSC is a cross-national World Health Organization (WHO) collaborative study that has been performed every fourth year since the 1980s. It includes standard questionnaires with mandatory questions used by all the participating countries to enable cross-national comparisons. In addition, there are optional packages enabling analyses of specific topics, as well as national, country-specific items [39, 40].

For the Swedish 2017/18 survey, a two-step cluster sampling was performed. First, a random sample of schools were selected. Then a class was randomly selected in school. All students in that class were invited to participate. The students completed the questionnaires with paper and pencil in their classrooms. Upon completion, they handed over the questionnaires to the teacher in sealed envelopes without any personal identification information, and the questionnaires were subsequently sent to Statistics Sweden. The students remained anonymous and were informed that participation was voluntary. In the 2017/18 survey, 4,294 students participated. The response rate was 47% at the school level, and 87%–90% at the student level [41]. The current study covers only students in grade nine (∼15–16 years) since the measure of mental wellbeing was included only in the questionnaires for this age group (*n* = 1,661). After exclusion of cases due to internal non-response (*n* = 243), the study sample included information from 1,418 students distributed across 78 classes. More information about the data collection is provided elsewhere [41].

### Measures

Mental wellbeing was operationalised by the Short Warwick-Edinburgh Mental Wellbeing Scale (SWEMWBS), transformed according to the conversion table presented by Stewart-Brown et al. [42]. The skewness of the (converted) index was 0.21 (boys: 0.17; girls: 0.23) and the kurtosis 2.93 (boys: 2.54; girls: 3.35). The SWEMWBS has been validated in samples of adolescents in several countries including England and Scotland [14], Ireland [32], Australia [17] and Norway [43]. The SWEMWBS is not included in the HBSC protocol, but was added to the Nordic countries’ questionnaires of the HBSC 2017/18 survey as part of a Nordic research collaboration on positive health [44].

School demands were constructed from three items. The same, or similar, items have been used in previous studies to capture school demands [7, 10, 21, 27, 29, 30]. While the question on feeling pressured by schoolwork is mandatory in the HBSC study, the other two items are optional and therefore included in the questionnaire in only some of the countries participating in the HBSC.

Teacher support was based on the Teacher Support Scale [45, 46]. Classmate support was based on the Classmate Support Scale [45, 46].

See Table 1 for more detailed information about the above-mentioned scales.

**TABLE 1:** Description of the measures of mental wellbeing, school demands, teacher support and classmate support. Swedish Health Behaviour in School-aged Children study 2017/18. (Sweden. 2017/2018).

Scale	Items	Response categories	Original index range	Truncated index range	Eigenvalue	Factor loadings	Cronbach’s α
Mental wellbeing (SWEMWBS)	**a.** “I’ve been feeling optimistic about the future”	**a-g**.	7–35^a^	-	3.86	0.67–0.82	0.90
	**b.** “I’ve been feeling useful”	1 = “None of the time”					
	**c.** “I’ve been feeling relaxed”	2 = “Rarely”					
	**d.** “I’ve been dealing with problems well”	3 = “Some of the time”					
	**e.** “I’ve been thinking clearly”	4 = “Often”					
	**f.** “I’ve been feeling close to other people”	5 = “All of the time”					
	**g.** “I’ve been able to make up my mind about things”						
School demands	**a.** “How pressured do you feel by the schoolwork you have to do?”	**a.**	3–14	5–12	1.26	0.55–0.71	0.72
	**b.** “I find the schoolwork difficult”	1 = “Not at all”					
	**c.** “The schoolwork makes me tired”	2 = “A little”					
		3 = “Some”					
		4 = “A lot”					
		**b-c**.					
		1 = “Almost never”					
		2 = “Seldom”					
		3 = “Sometimes”					
		4 = “Often”					
		5 = “Very often”					
Teacher support	**a.** “I feel that my teachers accept me as I am”	**a-c**.	3–15	8–15	1.95	0.79–0.83	0.86
	**b.** “I feel that my teachers care about me as a person”	1 = “Strongly disagree”					
	**c.** “I feel a lot of trust in my teachers”	2 = “Disagree”					
		3 = “Neither agree nor disagree”					
		4 = “Agree”					
		5 = “Strongly agree”					
Classmate support	**a.** “The students in my class(es) enjoy being together”	**a-c**.	3–15	8–15	1.65	0.69–0.78	0.82
	**b.** “Most of the students in my class(es) are kind and helpful”	1 = “Strongly disagree”					
	**c.** “Other students accept me as I am”	2 = “Disagree”					
		3 = “Neither agree nor disagree”					
		4 = “Agree”					
		5 = “Strongly agree”					

^a^ Transformed scale was used in the analyses (see Stewart-Brown et al. [42]).

Gender had the values 0 = “Boy” and 1 = “Girl.”

The Family Affluence Scale (FAS) was used to capture the family’s level of affluence, representing a proxy for socioeconomic status. Responses from six items were used to construct version III of FAS [47]: number of computers (0 = none, 1 = one, 2 = two, 3 = more than two); number of cars (0 = none, 1 = one, 2 = two or more); own bedroom (0 = no, 1 = yes); number of holidays abroad in the past year (0 = none, 1 = one, 2 = two, 3 = three or more); whether or not the family owns a dishwasher (0 = no, 1 = yes); and number of bathrooms (0 = none, 1 = one, 2 = two, 3 = three or more). Responses to these six items were summed to an index ranging between 0–13, with higher values indicating higher levels of affluence.

### Statistical Method and Analytical Strategy

All the analyses were run separately for boys and girls. Descriptive statistics were retrieved for the total sample and separately by gender. Differences were tested by means of t-tests.

We plotted the mean values of mental wellbeing across the scales of school demands, teacher support and classmate support, respectively (see Supplementary File). School demands had few very low and very high values, and the mean values of mental wellbeing were thus unstable at the low and high ends of the scale, potentially influencing the results of further analyses. Therefore, the values 3–4 of school demands were recoded as 5, and the values 13–14 as 12 (in total, 12% of the values were recoded). Teacher support and classmate support had few very low values, and the values 3-7 were recoded as 8 (8% and 6% of the values, respectively).

To examine the associations between psychosocial school conditions and mental wellbeing, we estimated a series of linear ordinary least squares regression models, using the truncated scales of school demands, teacher support, and classmate support. Since the students were clustered in school classes, robust standard errors were estimated using Stata’s “robust cluster” option. In Model 1, the crude association between school demands and mental wellbeing was assessed. In Model 2, the association was adjusted for family affluence, and in Model 3, teacher and classmate support were added. Gender differences in the associations between each of the independent variables on one hand, and mental wellbeing on the other hand, were assessed by linear regression models of the total sample that included an interaction term between the respective variable and gender. Models 4 and 5 included an interaction term of the continuous measure of school demands, with the continuous measure of teacher support and classmate support, respectively. The results of the interaction analyses were visualised by means of margins plots (adjusted predictions by Stata’s “margins” command). The analyses were performed with StataSE 16.1 [48].

## Results

Descriptive statistics of the study variables are presented in Table 2. On average, boys reported higher mental wellbeing than girls, as measured by the SWEMWBS. Girls experienced higher school demands than boys, on average, whereas boys on average experienced greater teacher and classmate support than girls. All gender differences were statistically significant. As expected, no gender difference in family affluence was observed.

**TABLE 2:** Description of the study sample. Swedish Health Behaviour in School-aged Children study 2017/18. (Sweden. 2017/2018).

	All (*n* = 1,418)		Boys (*n* = 660)		Girls (*n* = 758)		t-test
	Mean	s.d.	Mean	s.d.	Mean	s.d.	
Mental wellbeing (SWEMWBS)^a^	24.62	5.05	26.12	4.96	23.32	4.76	0.0000
School demands^b^	9.22	2.07	8.77	2.08	9.62	1.99	0.0000
Teacher support^c^	11.89	2.32	12.25	2.31	11.57	2.28	0.0000
Classmate support^c^	11.80	2.13	12.10	2.06	11.54	2.16	0.0000
Family Affluence Scale^d^	9.44	1.99	9.48	1.97	9.41	2.00	0.5183

^a^ Scale 7–35.

^b^ Truncated scale 5–12.

^c^ Truncated scale 8–15.

^d^ Scale 0–13.

Results from the linear regression analyses of mental wellbeing are presented in Table 3. In the unadjusted Model 1, school demands were negatively associated with mental wellbeing in both boys (b = −0.67, 95% CI −0.86, −0.48) and girls (b = −0.93, 95% CI −1.12, −0.75). FAS was positively associated with mental wellbeing but adjusting for FAS had almost no effect on the association between school demands and mental wellbeing (Model 2). In Model 3, positive associations with mental wellbeing were observed for both teacher support (boys: b = 0.53, 95% CI 0.34, 0.72; girls: b = 0.56, 95% CI 0.40, 0.72) and classmate support (boys: b = 0.70, 95% CI 0.50, 0.69; girls: b = 0.50, 95% CI 0.31, 0.69), while the estimate for school demands was closer to the null in this model, compared to previous models.

**TABLE 3:** Results from linear regression analyses. Predicted change in mental wellbeing per unit increase in the predictor variables, b coefficients and robust 95% confidence intervals (clustering for school class). Swedish Health Behaviour in School-aged Children study 2017/18. (Sweden. 2017/2018).

	Model 1	Model 2	Model 3	Model 4	Model 5
**Boys (*n* = 660)**					
School demands	−0.67 (−0.86, −0.48)	−0.67 (−0.87, −0.47)	−0.36 (−0.54, -0.19)^a^	0.65 (−0.50, 1.80)	1.11 (0.17, 2.04)
Teacher support			0.53 (0.34, 0.72)^b^	1.57 (0.72, 2.42)	
Classmate support			0.70 (0.50, 0.91)^b^		2.12 (1.46, 2.77)
Family Affluence Scale		0.45 (0.27, 0.63)	0.35 (0.18, 0.51)^b^	0.42 (0.24, 0.59)	0.35 (0.18, 0.51)
School demands*teacher support				−0.09 (−0.18, 0.00)	
School demands*classmate support					−0.13 (−0.20, −0.06)
Intercept	32.00 (30.25, 33.73)	27.73 (25.19, 30.26)	10.97 (7.46, 14.47)	6.47 (−4.91, 17.85)	1.14 (−7.35, 9.63)
R^2^	0.0790	0.1108	0.2988	0.2373	0.2634
**Girls (*n* = 758)**					
School demands	−0.93 (−1.12, −0.75)	−0.92 (−1.11, −0.74)	−0.65 (−0.79, -0.50)^a^	0.17 (−0.60, 0.93)	0.56 (−0.24, 1.35)
Teacher support			0.56 (0.40, 0.72)^b^	1.44 (0.77, 2.11)	
Classmate support			0.50 (0.31, 0.69)^b^		1.81 (1.13, 2.49)
Family Affluence Scale		0.34 (0.17, 0.51)	0.22 (0.07, 0.38)^b^	0.30 (0.14, 0.46)	0.23 (0.07, 0.38)
School demands*teacher support				−0.07 (−0.14, −0.01)	
School demands*classmate support					−0.11 (−0.18, −0.04)
Intercept	32.29 (30.45, 34.14)	29.03 (26.73, 31.33)	15.21 (11.98, 18.43)	10.21 (2.11, 18.30)	7.40 (−0.87, 15.69)
R^2^	0.1515	0.1716	0.3279	0.2912	0.2813

^a^ Gender difference statistically significant at *p* < 0.05.

^b^ Gender difference not statistically significant at *p* < 0.05.

**FIGURE 1:**
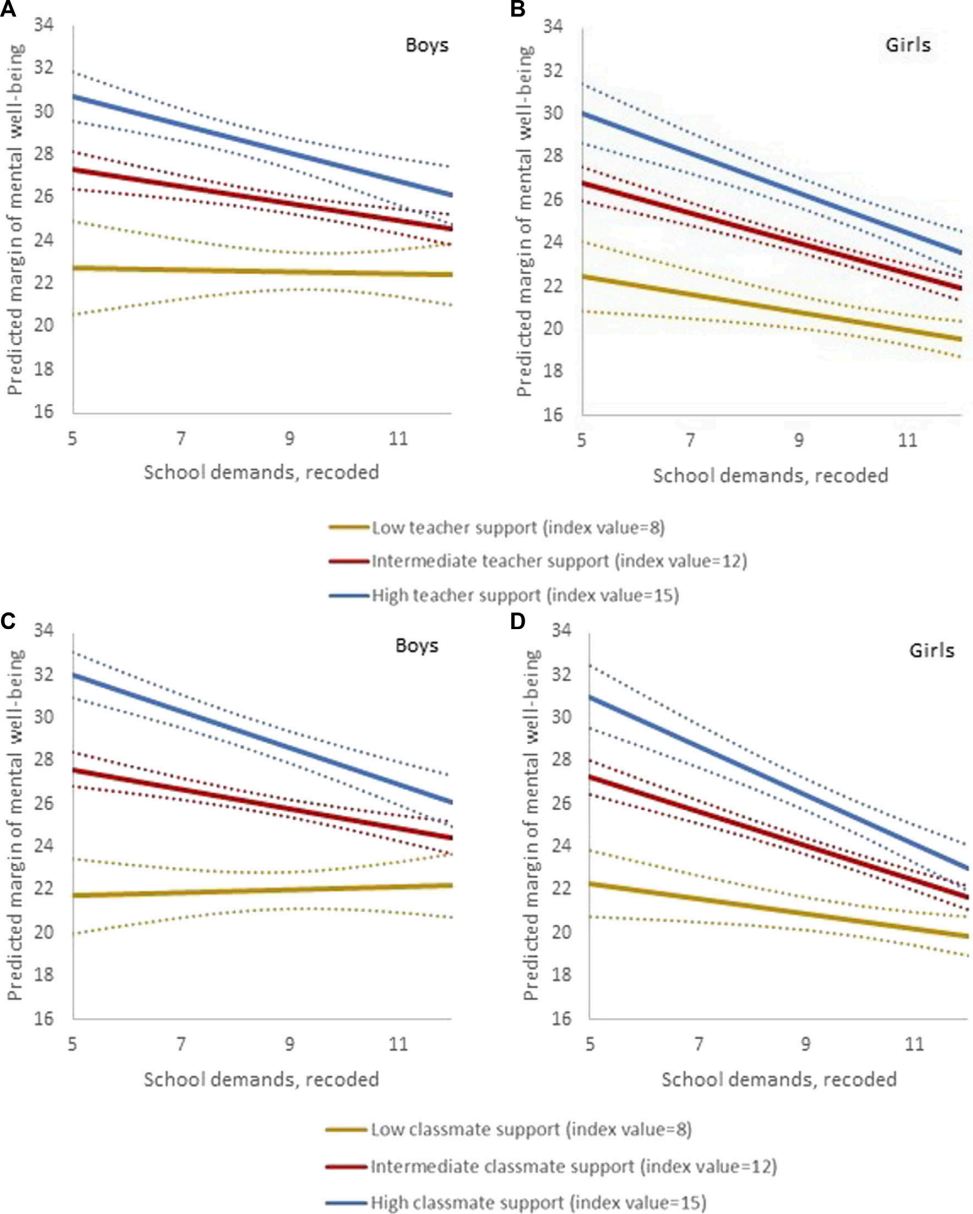
Visualisation of the interaction between school demands and teacher support, and school demands and classmate support, in gender-stratified linear regression models predicting mental wellbeing. The association between school demands and mental wellbeing is plotted at different levels of teacher support **(A,B)** and of classmate support **(C,D)** in boys (*n* = 660, left) and girls (*n* = 758, right). Dotted lines indicate 95% confidence intervals. Swedish Health Behaviour in School-aged Children study 2017/18. (Sweden. 2017/2018).

Models 4 and 5 included interaction terms of school demands with teacher support and classmate support, respectively. Figure 1 illustrates the interaction effects, depicting the association between school demands and mental wellbeing at low (index value 8), intermediate (at the median of the sample, index value 12) and high (index value 15) levels of teacher support (Figures 1A, B) and of classmate support (Figures 1C, D). In both boys and girls, higher levels of teacher support and classmate support were associated with higher overall levels of mental wellbeing. The negative interaction terms indicated an inverse association between school demands and mental wellbeing for both genders that was weaker at low levels of the support variables and became stronger with increasing levels of the respective support variables.

All the regression analyses were performed using the truncated indices of demands, teacher support and classmate support. As a sensitivity check, we also ran analyses using the original indices (data not shown). The results were similar to those presented, however the R^2^ in these models tended to be lower and the coefficients closer to the null. As another sensitivity check, we performed the same analyses as those presented in Models 4 and 5 whilst including both teacher and classmate support simultaneously (not shown). The results of the interaction analyses in these models were very similar to those presented in Table 3.

One of the items in the SWEMWBS, “I’ve been feeling close to other people”, relates to the respondent’s social relations with other persons and may thus be conflated with teacher and classmate support. However, pairwise correlations with the item “I’ve been feeling close to other people” were only moderate for teacher support (r = 0.38) and classmate support (r = 0.36). We also performed a sensitivity analysis where we omitted the item from the mental wellbeing measure (producing a scale ranging between 6-30). The results were similar to those reported (data not presented), indicating that the associations were not driven by this particular item.

Analyses of the total sample, including interaction terms between gender and each of the independent variables (not presented in table), showed that the negative association between school demands and mental wellbeing was statistically significantly stronger among girls than boys, while the associations between the other independent variables (FAS, teacher support, and classmate support) and mental wellbeing did not differ by gender.

## Discussion

Focusing on indicators of psychosocial school conditions as possible determinants of positive mental health among students, this study showed that, overall, school demands were negatively, and support from teachers and classmates positively associated with mental wellbeing among both boys and girls. We furthermore observed two interaction effects, which both pointed to a negative association between perceived demands and mental wellbeing at high levels of support, but to a correspondingly weak or non-existent association at lower levels of support. Thus, at higher levels of perceived teacher- or classmate support, the average decrease in mental wellbeing of experiencing high demands was markedly steeper than at lower levels of such support. This finding does not align well with the theoretical idea that social support can buffer against the link between perceived demands and health [5, 10]. However, the results showed that low social support was in itself clearly associated with poorer wellbeing, in line with the main effect model of social support [49]. According to this theoretical model, social support enhances wellbeing since it provides positive affect as well as a sense of predictability and stability, and also promotes feelings of self-worth [49]. Following this reasoning, it is also likely that low levels of social support may be stressful in themselves. One interpretation of our finding that school demands were more strongly linked with wellbeing at higher levels of support, is that the experience of low social support may “overshadow” the potential additional impact of a high burden of school demands.

A number of prior studies have reported that greater school demands are associated with more health complaints [3–12, 19, 20]. This study demonstrated that there were clear associations also with mental wellbeing, as one aspect of positive health, with higher demands being linked with lower wellbeing. Furthermore, school demands were more strongly and negatively associated with mental wellbeing among girls than among boys. This result reflects previous findings in relation to health complaints [8, 9, 12, 19, 23, 34]. In other words, the association seems to be consistent across different types of outcomes, suggesting that school demands seem to be more important for girls’ stress-related ill-health as well as their mental wellbeing. One interpretation of this pattern is that girls, due to gendered norms, tend to place greater emphasis on schoolwork and performance than boys [19], and that school demands therefore have greater implications for both their health complaints and mental wellbeing. Girls also reported greater school demands compared with boys. Given girls’ on average higher school performance, this result may be somewhat surprising but is, again, in accordance with earlier research [19, 21, 23, 27, 33]. Also, this finding may be interpreted in light of gendered aspirations. In other words, it is possible that girls on average perceive greater school demands than boys because they, on average, tend to aim higher in terms of their school performance [19, 50].

The associations that were shown between teacher and classmate support and mental wellbeing were not surprising. Nonetheless, they add to the existing body of research by showing that support from teachers and classmates are associated not only with fewer health complaints [5, 7, 10, 12, 21–24] but also with higher levels of positive mental health. No gender differences were found in the associations between teacher and classmate support and mental wellbeing, indicating that these types of social relations are equally important for the wellbeing of boys and girls. However, boys reported higher average levels of teacher and classmate support than girls.

Although family affluence was only a control variable in the present study, the finding that it was positively associated with mental wellbeing deserves to be highlighted. A previous study based on Danish HBSC data reported differences in low, but not in high, mental wellbeing by parents’ occupational class [51]. Future studies should examine whether social health inequities in positive mental health among adolescents exist also when using other indicators of socioeconomic status, such as parents’ education or income, preferably based on information collected from parents or from official registers.

The main strength of this study is the use of a measure of mental wellbeing that has been previously validated in similar age groups in various country settings [14, 17, 32, 43]. Another benefit is the relatively large sample size. However, there are also limitations. First, we acknowledge the difficulties in drawing conclusions about the size of the (statistical) effects of school-related conditions on mental wellbeing and about the qualitative implications of our findings for policymakers and stakeholders. Since our measure of mental wellbeing was based on a self-reported and subjective scale rather than a clinical measure, it is a delicate issue to define values that indicate an absolute “high” or “low” level of mental wellbeing. The same is true for the measures of school demands and support. In other words, we examined the general patterns between psychosocial school conditions and mental wellbeing, but are inhibited to make any claims in more absolute or practical terms with support in the data. To do this, other types of measures and/or another research approach (e.g., studying the effects of a specific intervention, or applying qualitative methods) would have been needed. Furthermore, the cross-sectional nature of the data inhibits causal explanations with support in the data. Although it seems reasonable to assume that students’ perceptions of school demands and social support may affect their mental wellbeing, it cannot be ruled out that the emotional state of students impacts on their perception of their psychosocial school conditions. To disentangle the directionality of the associations, future research should assess the links between psychosocial school conditions and mental wellbeing by use of longitudinal data. Another limitation concerns the possible systematic bias related to the attrition in two steps: at the school-level, a number of the sampled schools did not participate; and at the student-level, 13% of the ninth graders did not participate due to absence on the day of the survey [41]. Furthermore, since information is lacking about which schools in the sample that participated and which did not, there are limited possibilities of estimating the response bias at the school-level. With regards to student non-response, there may be a risk of systematic bias. One can assume that students who were absent due to truancy or illness have on average lower mental wellbeing, and possibly also experience greater school demands and less teacher and classmate support than students who took part in the study. However, we do not see any reasons why this possible bias would affect the associations found to any substantial degree; if anything, we believe that they may have been underestimated rather than the other way around.

Finally, it should be underlined that the current study was based on information collected among ninth grade students in Sweden. Grade nine is the final year of compulsory school, and academic performance is especially critical during this school year as the applications for upper secondary school are based on the student’s final grades from compulsory school. Thus, given the particular importance of school performance in grade 9, it is possible that school-related conditions are particularly strongly associated with student wellbeing (and adverse health) during this school year. Consequently, assessing the generalisability of the associations between school demands, teacher support, and classmate support and mental wellbeing requires data collected among students in other age groups and in other school systems.

### Conclusion

This study contributes with knowledge about how psychosocial conditions in school may hinder or enhance positive mental health among students. The analyses showed that mental wellbeing increased with greater teacher support and classmate support among both boys and girls. Higher school demands were associated with lower mental wellbeing among students experiencing higher levels of support, while the level of school demands did not have a strong association with mental wellbeing when teacher and classmate support were reported as low. Based on the study’s findings, it is possible to suggest that in order to promote students’ mental wellbeing, schools should support their students so that they feel that the demands are manageable, ascertain that their teachers have the time and possibilities to provide adequate support to their students, and enhance a positive social climate among students.
