# Supplementary material for: Psychosocial School Conditions and Mental Wellbeing Among Mid-adolescents: Findings From the 2017/18 Swedish HBSC Study
Source: Int J Public Health. 2023 Jan 5;67:1605167. doi: 10.3389/ijph.2022.1605167 (PMC9849233; doi:10.3389/ijph.2022.1605167)

Appendix 1 Mean values of mental well-being plotted across scales of school demands, teacher support and classmate support. Swedish Health Behaviour in School-aged Children study 2017/18.

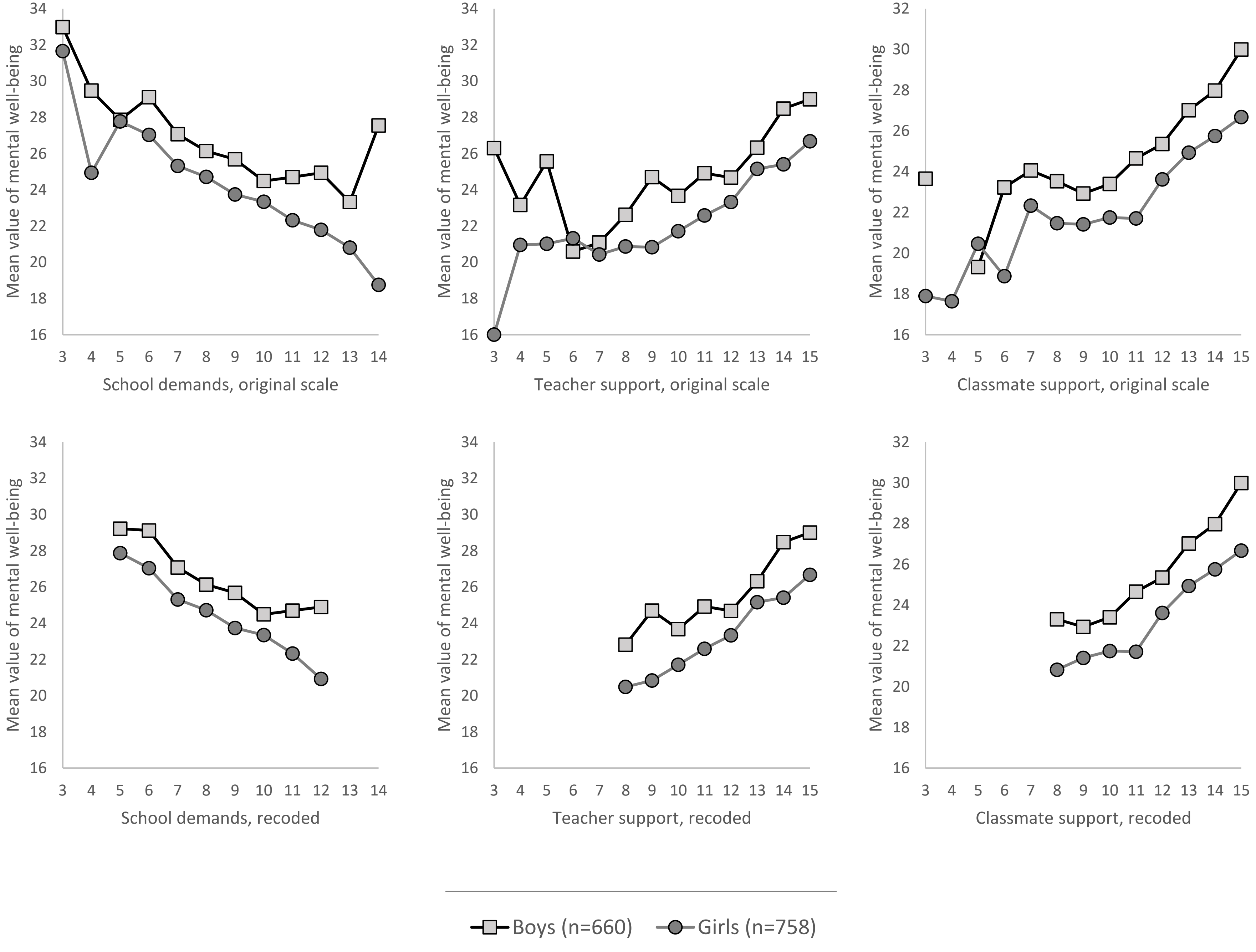

Supplement: Supplementary file 1 [file DataSheet1.PDF]
